# Favourable Changes of the Risk-Benefit Ratio in Alpine Skiing

**DOI:** 10.3390/ijerph120606092

**Published:** 2015-05-29

**Authors:** Martin Burtscher, Gerhard Ruedl

**Affiliations:** Department of Sport Science, Medical Section, University of Innsbruck, Fürstenweg 185, A-6020 Innsbruck, Austria; E-Mail: Gerhard.Ruedl@uibk.ac.at

**Keywords:** alpine skiing, injury risk, health benefits

## Abstract

During the past five decades recreational alpine skiing has become increasingly safer. The numerous annual media reports on ski injuries have to be interpreted on the basis of the tremendous numbers of skiers. These favourable changes seem primarily be due to the introduction of short carving skis, more rigid and comfortable ski boots, the use of protective gear like helmets, and the optimized preparation of ski slopes. The associated health benefits from skiing, especially arising from its association with a healthier life style, and possibly also from effects related to hypoxia preconditioning and increasing subjective vitality by natural elements clearly outweigh the health hazards. Technical improvements will likely help further reducing the injury risk. At least hypothetically, each individual skier could help to prevent injuries by the development of an appropriate physical fitness and responsible behaviour on ski slopes thereby optimizing the risk-benefit ratio of alpine skiing.

Epidemiological studies indicate that recreational alpine skiing has become increasingly safer during the past five decades but ongoing media coverage during the winter seasons seems not to confirm these facts. In addition, there are practically no reports that consider potentially beneficial health effects of downhill skiing. Thus, this commentary aimed at identifying the actual risk and pointing out potentially favourable health effect associated with downhill skiing.

## 1. The Risk

Daily press releases on skiing injuries during the winter seasons convey the impression of downhill skiing as a dangerous activity. In fact, these reports have to be interpreted in the light of the very large participation rates. Worldwide, there are more than 2000 downhill ski areas spread across 80 countries with an estimated 400 million skier days annually [1]. Assuming a death rate of 1 per 1 million skier days [2] and an injury rate of 1–2 per 1000 skier days [3,4] a total of 400 fatalities and 400,000 to 800,000 injured skiers would result, enough to fill newspapers daily, but actually representing a relatively low death and injury risk. Whereas the number of dead and injured skiers (needing medical treatment) and the location of injured body parts are usually collected by ski patrol members, information on the severity of injuries are provided by hospital emergency departments. The injury risk during downhill skiing is clearly lower when compared to soccer or other team sports but is comparable to that of cycling, running or tennis [5]. The death risk during mountain hiking in the Alps is about 5 times and during ice and rock climbing in the Alps or trekking in Nepal about 10 times higher than during downhill skiing [6]. In other words, if somebody skis for 20 days per winter season on average 1 death would occur within 50,000 years and 1 injury within 25 to 50 years. The death rate of about 1 per 1 million skier days (including traumatic and non-traumatic deaths) has remained relatively stable over the past five decades [2]. The vast majority of these fatalities are due to a collisions with a solid object such as trees or rocks [2] and would be mostly avoidable by choosing a skiing velocity appropriate for individual skiing skills. In contrast, the injury risk in downhill skiers has changed dramatically during the last six decades. For example, an epidemiologic study performed in 1961 at Mount Snow in Vermont found an overall injury rate of 5.9 per 1000 skier days [7] and a similar study conducted thirty years later at Blackcomb Mountain in British Columbia reported an injury rate of 2.9 per 1000 skier days [8]. Similar as mentioned above, the recorded number of injured skiers in these studies was based on ski patrol and/or the resort’s medical facility reports. The authors assumed, as we do for the most recent studies, that at least 95% of all injured skiers have been included for a given observation period.

Again, about 30 years later the newest data collected in large Alpine ski regions revealed a further drop of this rate to about 0.6 to 1.0 per 1000 skier days [3,9] but an injury rate of about 2.0 per 1000 skier days was reported from other regions [4]. Lower leg injuries decreased by about 90% since the introduction of release bindings in the 1960 s’ accounting for only 5%–7% in adult skiers but still exceeding 20% in children up to 12 years [3,10,11]. However, knee injuries, particularly tears of the anterior cruciate ligament (ACL) have become the most frequent skiing injuries, accounting for about 30% in males and more than 50% in females [3,4,12]. 

Injury rates vary largely with age, sex, and skiing ability as already demonstrated by the authors of the early Mount Snow Study [7]. In addition, a recent study demonstrated that activities performed in snow-parks may increase the risk of sustaining a severe injury compared with participation on other slopes [13]. Thus, differences between skier populations and variable slope conditions may explain different injury rates reported from different ski areas. 

## 2. Potential Causes for Risk Changes

The risk reduction during the first three decades (1960–1990) has primarily been attributed to the development of release bindings and appropriate ski boots [10]. The drop within the last three decades seems to be closely linked with the introduction of shorter skis (carving skis) and steady improvements of additional skiing equipment and ski slope preparation [3,4]. It has to be noted that this may not be true for competitive skiers who still have high injury rates, especially with regard to ACL injuries [14]. Knee injuries are typically caused by a critical load to the knee during falling [11]. Release bindings have primarily been designed to prevent tibia and ankle fractures by releasing horizontally at the toe and vertically at the heel, but most of the current bindings do not affect knee injuries [11]. The main reason for the stagnation of binding design in the last 20 years and the rigidity of recommendations for binding settings is the fear of an inadvertent release associated with risk of injury [11]. In view of the extraordinary frequent knee injuries in females and lower leg injuries in children however, actual recommendations for binding settings should urgently be reconsidered for these populations. An additional promising option might soon become reality by the implementation of “multidirectional” release bindings offering significant protection to the knee ligaments in the case of falls [11]. The steady reduction of injury rates during the last three decades is clearly against the anticipated trend of increased injury risk in alpine skiing associated with the introduction of carving skis [3]. In contrast, less aggressive short carving skis with a broader tip are much easier to steer than the traditional long skis leading to rapid improvement of skiing skills associated with a lower falling and injury risk [3]. Of course, this might be not the only reason for the drop in skiing injury rates. For instance, ski boots have become more rigid, thereby particularly reducing ankle injuries, but they also have become more comfortable during the last years making it easier to ski and likely preventing rapid fatiguing. The use of protective gear like helmets has increased and may contribute to injury reduction. For example, a recently published meta-analysis showed a 35% reduction of the general head injury risk and a 59% reduction for children younger than 13 years when using a ski helmet [15]. Also the preparation of ski slopes has improved in many ski areas which turned out to be one source explaining the relatively broad range of injury rates observed between individual ski regions [3]. Not surprising that the injury risk increases with the proportion of steep and ungroomed slopes and it is the decision of the individual skier to be prepared to use these slopes [16]. 

## 3. Potential Benefits

In contrast to many studies that dealt with the risk of traumatic and non-traumatic events associated with alpine skiing, data on favourable health effects are scarce [17]. Of course, one must not play down the non-traumatic risk, e.g., for sudden cardiac death or aortic dissection in a high risk sub-population of downhill skiers [18,19]. However, there is ample evidence that regular physical activity reduces morbidity and mortality, predominantly those arising from cardiovascular and metabolic diseases [20]. Alpine skiing undoubtedly contributes to the overall amount of individual physical activity and its beneficial effects on healthy aging. In addition, the exposure to moderate altitude where skiing activities are typically performed may support these favourable effects [21]. It also has been suggested that higher levels of leisure-time exercise are generally associated with a healthier lifestyle [22]. In fact, we recently demonstrated that longterm skiers showed more favourable lifestyle characteristics and a better health status compared to the general population [17]. Prevalences of known hypercholesterolemia, systemic hypertension, diabetes, the frequency of mental stress and the occurrence of memory deficits declined with increasing yearly skiing frequency [17]. Another study group also demonstrated that alpine skiing has the potential to improve cardiovascular risk factors and may represent an effective intervention for combating sarcopenia and weakness in old age [23,24].

In addition, alpine skiing at moderate or high altitude may represent a unique model of hypoxia preconditioning (HP) and might therefore evoke beneficial effects from HP under certain conditions [25]. Skiers perform repeated downhill runs of a several minutes duration using ski lifts or cable cars to ascend repeatedly from lower to higher altitude. At about 2000 m arterial oxygen saturation values remain relatively high during rest (>90%) but drop below 85% during short physical activities, especially on the first day at altitude [26]. Hypoxia has been used as a preconditioning stimulus since the 1990s and has emerged as a safe and easily applied stimulus which can minimize injury risk in several organs such as the heart [27]. The protective effects of HP result from a single exposure or several short episodes (2 to 10 min) of hypoxia [28]. Protection occurs immediately (early protection), lasting for a few hours, or delayed (late protection after a vulnerable phase of about 24 h), lasting for several days [25,28]. In fact, analyses of our data on sudden cardiac deaths in male downhill skiers were shown to be consistent with an episode of early protection for about three hours and a subsequent vulnerable episode during the first skiing day at altitudes at or above 1700 m. Thus, we concluded that skiers at risk might actually benefit from HP but should avoid skiing longer than three hours on the first day at altitudes above 1700 m [25]. Last but not least, positive relationships have also been reported simply between being outdoors and subjective vitality mediated by the presence of natural elements related to alpine skiing like mountains, snow, wind, *etc.* [29]. Taken together, alpine skiing has become increasingly safer during the past five decades. 

In conclusion, the numerous annual media reports on ski injuries have to be interpreted on the basis of the tremendous numbers of skiers. In our opinion, the associated health benefits from skiing, especially arising from its association with a healthier life style, possibly also from effects related to hypoxia preconditioning, and increasing subjective vitality by natural elements clearly outweigh the health hazards. Technical improvements will likely help further reducing the injury risk. At least hypothetically, each individual skier could help to prevent injuries by the development of an appropriate physical fitness and responsible behaviour on ski slopes thereby optimizing the risk-benefit ratio of alpine skiing.
